# Design of a Compact Ultra-Wideband Bio-Inspired Antenna Based on the Antennal Structure of *Allomyrina dichotoma*

**DOI:** 10.3390/biomimetics11090606

**Published:** 2026-08-25

**Authors:** Xu Zheng, Chaobo Li, Chenxi Gao

**Affiliations:** 1 Institute of Microelectronics of the Chinese Academy of Sciences, Beijing 100029, China; zhengxu@ime.ac.cn (X.Z.);; 2 University of Chinese Academy of Sciences, Beijing 100049, China

**Keywords:** *Allomyrina dichotoma*, structural biomimetics, bio-inspired antenna, ultra-wideband, miniaturized antenna, rhinoceros beetle antenna, multi-segmented structure, biomimetic design

## Abstract

Grounded in structural biomimetics, this study extracts the multi-segmented tapered geometry from the 10-segmented lamellate antenna of *Allomyrina dichotoma* and maps it to microwave antenna design. Through biological characterization and parametric modeling, key geometric features—multi-segmented configuration, irregular contour, and bilateral symmetry—were extracted. Along the 2D pathway, a 10 × 10 × 1 mm^3^ PCB microstrip antenna was designed and fabricated, achieving 111% fractional bandwidth from 4.86 to 17.05 GHz with a peak gain of 2.15 dBi and a radiation efficiency of 67–72% across the operating band, plus two additional bands at 24.76–28.58 GHz and 32.66–37.73 GHz. The multiple resonance valleys on S_11_ curves and frequency-dependent surface current evolution indicate that multi-mode resonant coupling, perimeter increment, and symmetric aperture efficiency together underpin the ultra-wideband performance. Along the 3D pathway, a dipole antenna replicated via metallic 3D printing attains an electrical length of 0.17λ, with 66% bandwidth and 1.45 dBi gain, confirming the same geometric principle in a shape-preserving form. The 2D route favors planar integration and bandwidth, while the 3D route offers extreme miniaturization. This work provides experimental validation of cross-domain geometric mapping from biology to electromagnetics within structural biomimetics, and offers engineering evidence for the intrinsic versatility of this morphology across physical domains.

## 1. Introduction

Recent advances in portable IoT devices, miniature unmanned systems, and wearable electronics have imposed strict compactness and broadband requirements on microwave RF front-end hardware. While microstrip patch antennas offer low-profile geometry and superior integration compatibility, their inherent narrowband characteristic (bandwidth typically under 5%) severely limits practical application scenarios. This bottleneck arises from the simple current flow paths and limited resonant modes of conventional regular patch structures, which make it difficult to balance bandwidth expansion, gain performance, radiation stability, and device miniaturization through traditional design strategies [1]. Widely adopted broadband improvement methods, including parasitic loading, aperture coupling, and defected ground structures, can optimize bandwidth response yet tend to enlarge antenna size or complicate fabrication [2,3]. Such compromises inevitably create a conflicting relationship between miniaturization and broadband capability in real engineering designs [4].

Bio-inspired engineering provides a viable solution to resolve the inherent trade-off between antenna miniaturization and broadband performance [5]. Natural creatures have evolved sophisticated and optimized morphologies over prolonged evolutionary processes, offering plentiful innovative structural prototypes for engineering design. In antenna research, biomimetic methods exploit naturally optimized geometries and functional characteristics to break through the performance bottlenecks of conventional artificial antenna structures [6,7]. A review reported by Azam comprehensively summarized recent advances in bio-inspired antenna development. The literature verified that biomimetic antennas exhibit superior overall performance from microwave to terahertz bands, validating their feasibility as an effective alternative to conventional design schemes [8]. Existing bio-inspired antenna prototypes adopt diversified natural morphologies, including fractal structures abstracted from snowflakes, tree branches, cloud textures, and butterfly wings, as well as contour features extracted from plant leaves and bird feathers [9,10,11].

Biomimetics, however, operates at multiple levels. Functional biomimicry asks what a biological system does—for instance, how insect antennae detect chemical signals—a question that has long been central to biological research. Structural biomimicry, by contrast, looks at what biological forms themselves can offer to engineering design. Evolution is, in essence, a rigorous multi-objective optimization process: every morphological feature represents a “solution” refined under competing constraints—mechanical stability, material economy, spatial efficiency, and functional performance—over millions of years. Biological forms therefore embody engineering principles that transcend any single function. Their geometric logic often proves transferable across physical domains. Structural biomimicry starts precisely from this premise—extracting those geometric principles and adapting them to engineering problems. This study falls within the structural biomimetic category. Yet structural biomimicry is not separate from its functional counterpart: when engineering research validates the cross-domain effectiveness of a given morphology, it simultaneously offers a new line of evidence for understanding why that morphology was selected by evolution in the first place. Examples of this approach are already present in the literature on two-dimensional bio-inspired antennas, which has drawn heavily from plant-based structures, including leaf outlines, petal contours, and fractal branching patterns. In a 2023 study published in *Biomimetics*, Lozano et al. systematically analyzed the leaf geometries of 100 plant species and applied the extracted morphological features to microstrip patch antenna design, demonstrating that plant-derived structures can serve as viable design templates for antennas [12].

Meanwhile, for flexible broadband antenna development, Tan et al. proposed a novel antenna inspired by the venation structure of holly leaves [13]. By engraving vein-shaped slots on the antenna patch to generate inter-slot capacitive coupling, the proposed antenna achieves a continuous 100% fractional bandwidth covering the 2.4–7.1 GHz frequency range. This bandwidth fully covers commercial communication bands including n41, n78, n79 and the 5.8 GHz ISM band, with a peak gain of 5.4 dBi. Similarly, Yu et al. developed a flexible multi-band antenna with a bionic spider-web structure on a 40 × 50 × 0.1 mm^3^ polyimide substrate, realizing three independent operating bands with fractional bandwidths of 42.4%, 16.2% and 12.5%, respectively [14]. Beyond these classic prototypes, natural morphologies including ginkgo leaves, daisy petals and banana leaves have also been explored for multi-band and broadband antenna design. Notably, fractal structures that mimic the self-similarity characteristics of natural snowflakes, tree branches, clouds and butterflies have been validated as an efficient solution for antenna miniaturization [15,16].

Different from planar bio-inspired antennas that rely on planar contour imitation, three-dimensional bio-inspired antenna designs focus on replicating the functional structural mechanisms of biological sensing, auditory and locomotive systems [17,18]. For example, the unique outer ear morphology of horseshoe bats has guided the design of a 3D horn-shaped ultra-wideband antenna operating at 3–10 GHz, which is applicable to 5G communication scenarios [19]. Researchers have also developed a broadband arched dipole antenna inspired by bird rib structures. This design combines symmetric arched dipole units with dual L-shaped monopoles, and adopts conformal dielectric loading to replicate the curved profile of biological ribs, thus realizing excellent broadband radiation performance [20]. In addition to morphological bionics, sensory bionic mechanisms also show great application potential. Antenna arrays designed based on the directional hearing principle of the parasitoid fly *Ormia ochracea* can break the directivity limitations of conventional array structures, achieving remarkable improvement in directional radiation performance [21,22]. Such research progress reveals a distinct development trend for bio-inspired antennas: bionic design theories and structures continuously break the performance limits of traditional antennas, promoting structural innovation from conventional planar layouts to advanced three-dimensional configurations.

Despite these achievements, critical research gaps still exist in current bio-inspired antenna studies [23,24]. In addition to the extensive work on plant-shaped bio-inspired antennas, preliminary efforts have also been made in the specific direction of insect antenna-inspired designs. Omar et al. (2024) drew inspiration from the serrated edge of beetle antennae and developed a compact 5G patch antenna, achieving an absolute bandwidth of 4.69 GHz and a gain of 4.677 dB within a 30 × 30 mm^2^ footprint [25]. These attempts have confirmed that insect antennal morphologies can serve as viable templates for antenna design. However, most of these studies remain at the level of replicating the superficial contours of insect antennae. They have addressed whether a given shape can be used in antenna design, but have not systematically investigated why a particular morphology enables broadband response. More specifically, the multi-segmented tapered geometry common to many insect antennae has not yet been explored as an independent design principle, nor has the way its graded dimensions shape the resonant behavior. Among various segmented natural structures, the rhinoceros beetle antenna offers a distinctive combination of features: a multi-segmented configuration with gradually tapering dimensions, bilateral symmetry, and a lamellate three-dimensional curvature. This combination is of particular interest for antenna design, as the gradual variation in segment sizes can potentially support a graded distribution of resonant frequencies, while the bilateral symmetry contributes to pattern stability. These considerations motivate our selection of this specific morphology as the biological template for the present study. In addition, few theoretical models can quantitatively correlate biological structural parameters with electromagnetic performance. Furthermore, there is a lack of comparative research on 2D and 3D antenna designs derived from identical biological prototypes [26,27]. Accordingly, how structural dimensionality regulates antenna radiation behavior and broadband characteristics has not been fully clarified.

Structural biomimetics starts from the premise that biological forms embody geometric principles that can be extracted and reapplied across different physical domains. Guided by this rationale, the present study adopts the antennal structure of as its biological template. The antenna of this insect is lamellate in shape, consisting of ten segments whose dimensions grade progressively along the longitudinal axis. From a geometric standpoint, this multi-segmented tapered configuration exhibits multi-modal vibration behavior in the mechanical domain: each segment, differing in size from the others, possesses a distinct natural frequency, which collectively enables a broad frequency response to vibrational stimuli. The central hypothesis of this work is that this geometric principle can be transferred to the electromagnetic domain: specifically, that the graded multi-segmented morphology can be translated into a multi-section patch antenna, where the dimensional variations among sections excite multiple adjacent resonances that couple to produce a continuous broadband response. The feasibility of such cross-domain mapping rests on the observation that geometric principles are not confined to a single physical context. The resulting structural features, segmented units that offer varying current path lengths, a tapering profile that allows controlled current density distribution, and bilateral symmetry that supports pattern stability are intrinsically electromagnetic. In practice, the multi-segmented layout functions as a set of coupled resonators, enabling broadband operation; the irregular, tapered contour extends the effective current path and increases the electrical length, thereby facilitating miniaturization; and the inherent symmetry helps suppress cross-polarization and improve gain. This analysis indicates that the choice of prototype is not arbitrary but grounded in structural biomimetic reasoning: the fact that this morphology has been conserved through evolution suggests its structural effectiveness under multiple physical constraints. This is precisely the logic of structural biomimetic design to identify geometric principles from biological forms and test their viability in a target engineering domain.

This work focuses on the extraction and cross-domain validation of geometric principles. The multi-segmented tapered geometry of the rhinoceros beetle antenna is first interpreted as a mechanical principle, one that supports multi-modal vibration in the mechanical domain. It is then mapped to the electromagnetic domain to achieve multi-mode resonance coupling. Through this mapping, we aim to show that the geometric morphology of a bio-inspired antenna prototype carries extractable functional value beyond its biological context.

To address the aforementioned research gaps, this paper introduces the segmented tapered morphology of the 10-segmented rhinoceros beetle antenna into antenna design. The work is conducted in three aspects: (1) high-precision biological characterization and parametric modeling are performed to extract key geometric features including the multi-segmented configuration, irregular contour, bilateral symmetry, and inter-segment continuity; (2) quantitative mapping models from geometric features to electromagnetic performance are established to elucidate the underlying mechanisms by which the multi-segmented irregular structure achieves miniaturization and ultra-wideband operation; and (3) a 10 × 10 × 1 mm^3^ two-dimensional PCB microstrip antenna and a three-dimensional metallic 3D-printed dipole antenna are designed and fabricated to experimentally validate the effectiveness of the proposed bio-inspired design methodology.

## 2. Materials and Methods

### 2.1. Biological Prototype Extraction and Structural Biomimetic Design

*Allomyrina dichotoma* possesses a typical lamellate antenna with strong bilateral symmetry and a total length of approximately 5 mm [28,29,30]. Following the established principles of biological specimen preparation and microstructural characterization, a specimen preparation, microstructural extraction, and scale modeling workflow was adopted to analyze one antenna of *A. dichotoma*.

An adult *A. dichotoma* specimen preserved in formalin solution was retrieved, and one antenna was excised from the head using surgical scissors. The excised antenna was immersed in a centrifuge tube containing 75% ethanol and subjected to ultrasonic cleaning for 50 s, repeated three times, to remove surface contaminants. The specimen was subjected to graded ethanol dehydration through a series of 80%, 85%, 90%, 95%, and 100% ethanol, with 30 min immersion at each concentration [31,32]. The absolute ethanol step was repeated three times. After dehydration, the specimen was dried using an automated critical point dryer (Leica EM CPD300,Leica Microsystems GmbH, Wetzlar, Germany) with 30 drying cycles at 120 s intervals. The dried specimen was then mounted onto a specimen stub, yielding an intact antenna sample of *Allomyrina dichotoma*.

**Figure 1:**
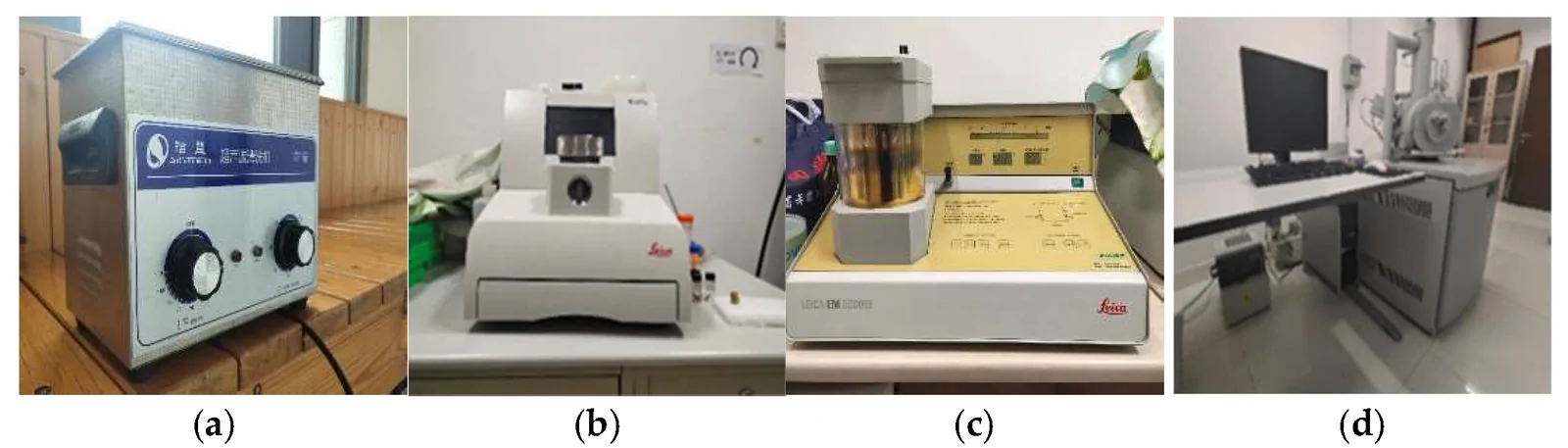
Equipment used for extracting the microstructure of the antennae: (**a**) Ultrasonic cleaning instrument. (**b**) Critical point dryer. (**c**) Advanced cold sputter coater. (**d**) Tungsten filament environmental scanning electron microscope.

Owing to the small size, lightweight, abundant sensilla, and complex internal structure of the antenna, conventional light microscopy could not adequately resolve its microstructure. To enhance surface conductivity and imaging contrast, the sample was sputter-coated with gold using a cold sputter coater (Leica EM SCD050, Leica Microsystems GmbH, Wetzlar, Germany) prior to SEM imaging (Figure 1). After gold deposition, the specimen provided sufficient contrast and resolution for observation under an FEI Quanta 450 environmental scanning electron microscope operated at 12.5 kV.

**Figure 2:**
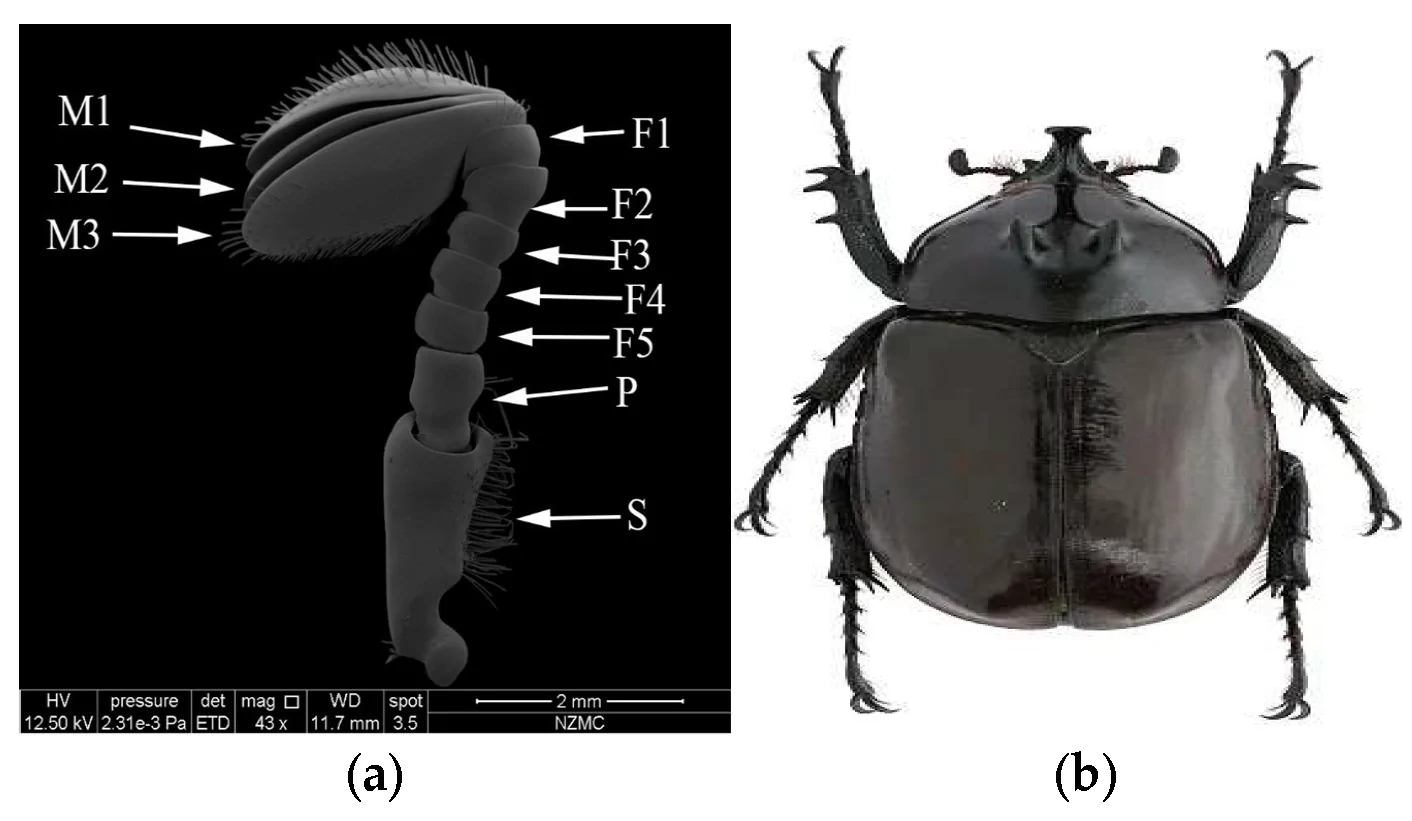
The external morphology and microstructural diagram of the antenna of *Allomyrina dichotoma*: (**a**) SEM image of the overall external morphology. (**b**) Photograph of an intact adult specimen of *Allomyrina dichotoma*.

SEM imaging in Figure 2a revealed that the lamellate antenna of *A. dichotoma* comprises the scape (S), pedicel (P), flagellum (F1-F5), and lamellar part (M1-M3). The flagellum is differentiated into three lamellae, which together form the lamellar part. The scape—the basal segment of the antenna—is a twisted tubular subsegment characterized by an irregularly cylindrical distal portion and a slender, short base. The flagellum consists of five subsegments, and its distal end bears three lamellae. Based on their proximity to the body, these three lamellae are designated the proximal lamella (M3), the medial lamella (M2), and the distal lamella (M1). In the resting state, the three lamellae are folded together, providing physical protection to the sensilla on their inner surfaces. When the insect needs to perceive external olfactory signals, the lamellae actively spread apart, exposing the sensilla to the environment. Due to the dehydration process during specimen preparation, the angle between the lamellar part and the flagellum in the prepared specimens is typically acute. In living *A. dichotoma*, however, this angle is a right angle in Figure 2b, and when the insect exhibits stress responses, the angle becomes obtuse. Apart from this angular change, the other morphological features of the antenna remain consistent with those of the living insect. Some sensilla distributed on the antenna have micrometer-scale dimensions (<10 um), which are much smaller than the operating wavelength of the antenna (approximately 60 mm at 5 GHz). Accordingly, these structures can be neglected in electromagnetic modeling.

### 2.2. Three-Dimensional Geometric Modeling and Bio-Inspired Dipole Antenna Design

#### 2.2.1. Three-Dimensional Geometric Modeling

Based on SEM imaging and optical microscopic measurements, each antennal segment appears approximately elliptical in two-dimensional projection, though with noticeable contour irregularities. To capture this irregularity quantitatively, we adopted the super ellipse formulation as known as the Gielis equation to parametrically fit the outline of each segment [33,34]. In polar coordinates, the boundary of the k-th segment is expressed as:

(1) $$r_{k}(\phi )={\left[{\left|\frac{1}{a_{k}}cos\left(\frac{m_{k}\phi }{4}\right)\right|}^{n_{k2}}+{\left|\frac{1}{b_{k}}sin\left(\frac{m_{k}\phi }{4}\right)\right|}^{n_{k3}}\right]}^{-1/n_{k1}}$$

Here, a_k_ and b_k_ are the lengths of the major and minor axes of the k-th segment (in mm), respectively, which were directly measured from the SEM images; m_k_ is the symmetry parameter, set to m_k_ = 4 for the bilaterally symmetric structure; and the shape exponents n_k1_, n_k2_, and n_k3_ control the curvature of the contour and were determined by fitting the measured profiles. Measurements show that the shape exponents of the segments of the *A. dichotoma* antenna range from 1.5 to 3.0, reflecting the degree of irregularity of the contour relative to a standard ellipse (for which n_k1_ = n_k2_ = n_k3_ = 2). Table 1 lists the measured values of a_k_, b_k_, and the shape exponents for each of the 10 segments.

The antennal axis is curved rather than a straight line in three-dimensional space [35]. To capture this feature, we defined the axis as a space curve:

(2) $$C(s)=[x(s),y(s),z(s){]}^{T}$$

Here, s denotes the arc length along the antennal axis (in mm). We reconstructed the axis path by measuring the projection coordinates of the antenna from multiple SEM viewing angles and applying cubic spline interpolation. The axis curvature is approximately 0.15 mm^−1^, and the path is nearly planar (torsion τ ≈ 0). The position of each segment center is determined by the arc length parameter s_k_, with consecutive centers evenly spaced along the axis according to measured segment intervals. Under bilateral symmetry, the left and right antennae are mirror images across the symmetry plane: if the right-side center has coordinates (x_k_, y_k_, z_k_), the corresponding left-side center is (x_k_, -y_k_, z_k_). We strictly preserved this symmetry in the 3D model to ensure the dipole antenna’s balanced radiation characteristics.

#### 2.2.2. Three-Dimensional Bio-Inspired Antenna Design

The antennae are naturally arranged as a pair of symmetrically distributed arm-like structures on the insect head, closely resembling the topology of a dipole. Given that the arm length substantially exceeds the arm width, the biological prototype’s three-dimensional configuration can be directly used for antenna construction without topological conversion. To explore the potential of this 3D bio-inspired design, we imported the previously established antennal model into simulation software for further modeling, with the junction at the base of the two arms serving as the feeding port. For subsequent fabrication and testing, we set the antenna material to pure copper with a relative permittivity of 1.

We used a rectangular lumped port as the feed, placing it between the bases of the two antennal arms. The port was initially 5 mm long and matched the base diameter (about 0.67 mm) in width. Excitation was set from one arm to the other, with an impedance of 50 Ω for compatibility with standard test equipment. The two arms are mirror-symmetric about the central plane, and the port lies on this symmetry axis to preserve balanced differential feeding. The antenna was modeled as pure copper (conductivity: 5.8 × 10^7^ S/m, relative permittivity: 1) to simplify the simulation and align with the subsequent 3D printing material.

We built the HFSS model using a terminal-driven solver with lumped-port excitation, and defined a rectangular air box surrounding the antenna as the radiation boundary. The frequency was swept from 3 to 7 GHz in 10 MHz steps. Initially, we set the angle α between the lamellar part and the flagellum to 90° to mimic the insect’s resting posture. With all other dimensions fixed, the feeding-port length *L*4 governs the antenna’s overall size and performance, so we performed a parametric sweep of *L*4 to examine the return-loss (S_11_) behavior. Because α varies in the living insect often adopting a right or obtuse angle, we also simulated the model with *L*4 fixed at 10 mm and *α* = 120°, evaluating both return loss and radiation pattern. The results showed that increasing α shifted the center frequency upward with a slight degradation in directivity. We therefore adopted the 90° configuration as the final design.

#### 2.2.3. Fabrication of the Three-Dimensional Bio-Inspired Antenna Samples

**Figure 3:**
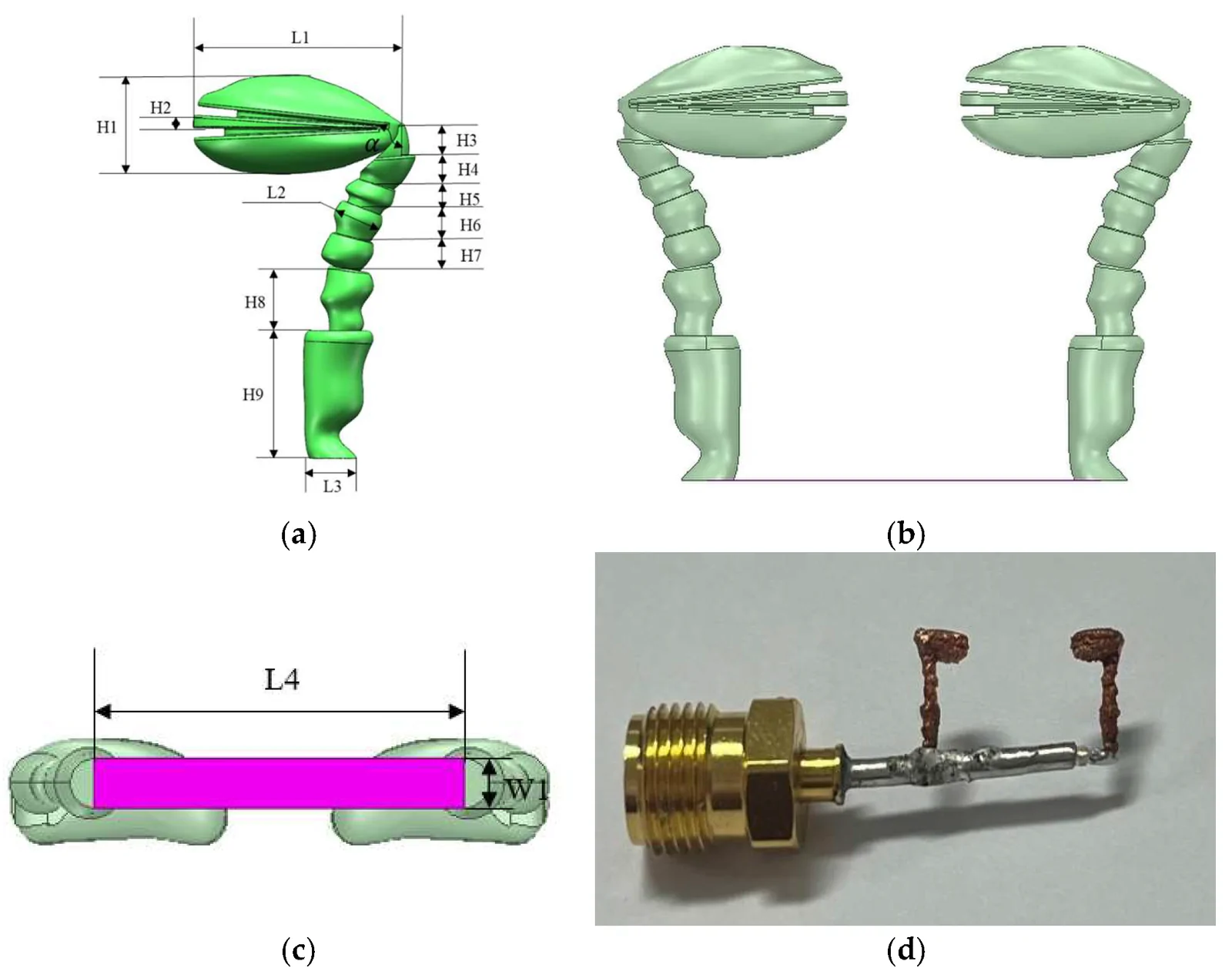
Design drawings of the three-dimensional bionic antenna: (**a**) Structural schematic of the 3D bionic antenna. (**b**) Front view of the dipole antenna model. (**c**) Bottom view of the dipole antenna model. (**d**) Photograph of the fabricated 3D bionic antenna prototype.

To assess fabrication feasibility, we exported the model in STEP format and printed it in pure copper using laser powder bed fusion (LPBF) with a 30 um layer thickness and ±0.1 mm accuracy, which preserved the main contour features of each segment. After printing, the samples were sandblasted to remove unfused powder. Given the small arm dimensions (total length ~5.6 mm) and the narrow root diameter (0.67 mm), direct soldering was challenging. To facilitate this, we reserved a cylindrical solder pad (0.67 mm in diameter, 1.5 mm long) at the root of each arm to increase the contact area. The two arms were then soldered to the inner and outer conductors of an SMA female connector, respectively, forming a balanced feed. A silver-based solder (melting point ~280 °C) was used to ensure both mechanical strength and electrical conductivity. After soldering, the joints were cleaned with alcohol to remove flux residues, and the inter-arm angle was calibrated to 90°. The final sample is shown in Figure 3d.

### 2.3. Design and Fabrication of the Two-Dimensional Ultra-Wideband Bio-Inspired Antenna

In contrast to the three-dimensional modeling, which requires precise replication of the biological prototype, the two-dimensional ultra-wideband bio-inspired antenna design aims to retain the key geometric features under planar fabrication constraints and map them to a manufacturable microstrip antenna structure. Accordingly, mathematical precision of the contour was not pursued. Instead, a regularization and equivalence treatment was applied to the biological contour while preserving the multi-segmented distribution, size tapering, and bilateral symmetry. The validity of this approach was subsequently verified by simulation and measurement results.

We projected the 3D antenna model orthogonally onto a 2D plane along its symmetry plane. This operation preserves the original multi-segment layout, irregular outline, and bilateral symmetry of the prototype, while eliminating all vertical dimensions. After projection, the narrow inter-segment necks were transformed into notch structures along the patch edges. These structural notches act as distributed capacitive elements in electromagnetic operation, which effectively adjusts resonant frequencies and broadens the impedance bandwidth. The projected biological contour was optimized iteratively through electromagnetic simulation and parametric tuning. Multiple targeted regularization strategies were implemented. Random irregular branches were standardized into regular rectangular and elliptical units to reduce structural disorder and facilitate physical fabrication. Meanwhile, the antenna radiator was stretched along the Z-axis to enlarge its radiation area and overall perimeter, extending the electrical length and lowering the operating frequency range. Additional slots were introduced at the junction of the two antenna branches to further improve the bandwidth and overall radiation performance. All uneven outlines of segmented sections were reconfigured using a neat assembly of ellipses, semi-ellipses, and rectangles. Smooth circular arcs were added at every segment joint to replicate the seamless transitional features seen on real insect antennae. After regularization, the design parameters of the patch were reduced from over 100 discrete coordinate points in the original projection to 20 independent variables in Table 2, including the widths (W1–W7), lengths (L1–L7), and angle parameters (α1–α6) of each segment. This design approach follows the research trajectory of existing two-dimensional bio-inspired antennas pursuing ultra-wideband characteristics. It aims to excite multiple adjacent resonant modes through the multi-segmented irregular contour. The dimensions of each segment vary gradually along the antennal axis, resulting in a gradient distribution of resonant frequencies that couple with each other to form a continuous wideband response.

**Figure 4:**
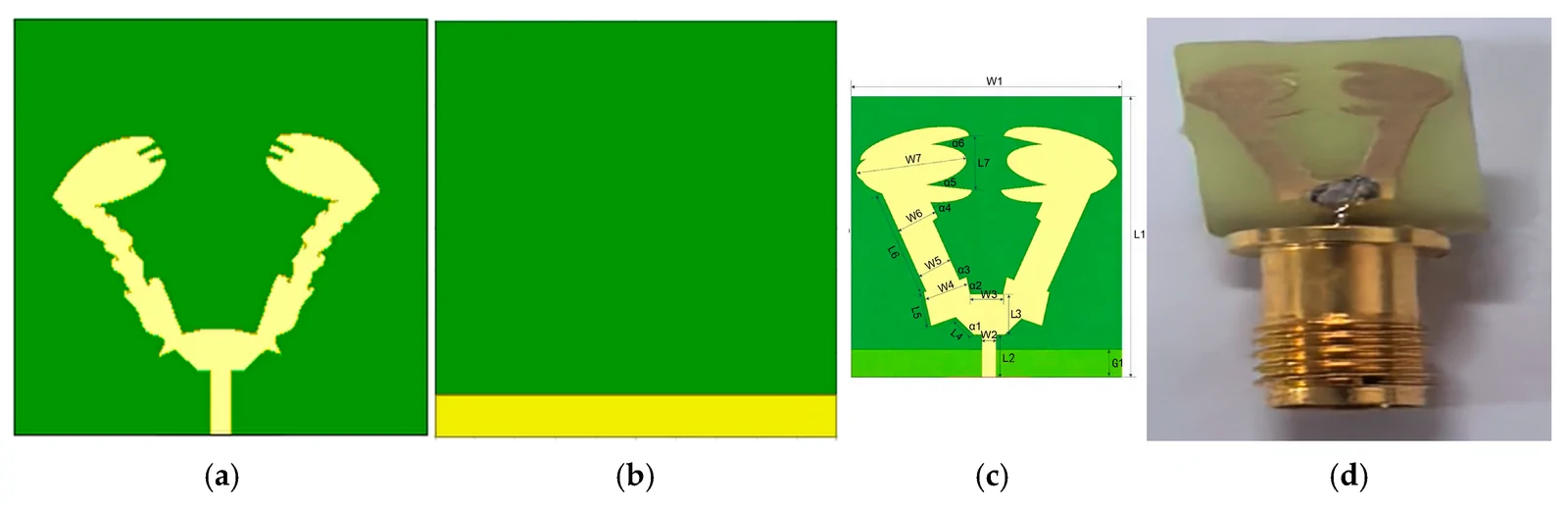
Schematic diagrams of the two-dimensional bionic antenna: (**a**) Front view of the simply mapped structure of the 2D bionic microstrip antenna. (**b**) Back view of the simply mapped structure of the 2D bionic microstrip antenna. (**c**) Schematic of the optimized 2D bionic microstrip antenna structure. (**d**) Photograph of the fabricated two-dimensional bionic microstrip antenna prototype.

The antenna uses a microstrip edge-feed configuration. A 0.5 mm-wide microstrip feed line extends from the standardized radiating patch to the substrate boundary, where it is soldered to the inner conductor of the SMA connector. The antenna is built on a 1 mm-thick FR-4 substrate (*εr* = 4.4, tan*δ* = 0.02) with 35 μm copper cladding on both sides. A compact 10 × 1 mm^2^ ground layer is printed on the substrate’s bottom surface and welded to the outer conductor of the SMA connector. Shrinking the ground footprint weakens capacitive coupling effects and suppresses intense cavity resonance that commonly arises in microstrip antennas with full ground planes, which in turn simplifies broadband impedance matching. The antenna adopts an edge-feeding scheme on the substrate. High-frequency signals travel along the microstrip line from the patch edge and return via the bottom ground plane, forming a complete closed radio-frequency transmission path. The finalized patch configuration is displayed in Figure 4c, with overall physical dimensions of 10 × 10 × 1 mm^3^.

The antenna was modeled in simulation software with a driven-terminal solver and a 1–40 GHz frequency sweep. Parametric sweeps were conducted on W1–W7, L1–L7, and α1–α6 to optimize S_11_ below −10 dB in the 5–17 GHz band. The search ranges for these parameters were derived from the biological measurements to maintain geometric similarity with the biological prototype: W1–W7 (0.5–10 mm), L1–L7 (1–10 mm), and α1–α6 (25–135°). The simulations employed adaptive mesh refinement with a convergence criterion of maximum Delta S < 0.02, typically converging within 10–15 iterations. Fabrication followed a standard PCB screen-printing process with an etching accuracy of ±0.05 mm, and the patch and feed line were chemically gold-plated to prevent oxidation. The SMA connector was soldered at the substrate edge, with the inner conductor connected to the top-side microstrip line and the outer conductor to the bottom ground plane. The fabricated sample is shown in Figure 4d. S-parameters were recorded using an Agilent VNA E8361C (Santa Clara, CA, USA), while gain and radiation patterns were measured in a microwave anechoic chamber (800 MHz–40 GHz). We calibrated the Agilent VNA before taking any measurements. A standard two-port SOLT procedure was used, with the reference plane set at the SMA connector. For the gain and radiation pattern tests in the anechoic chamber, we used the gain-comparison method with a standard gain horn as our reference. Taking into account the calibration accuracy, chamber reflections, cable stability, and the 8 m test distance, we estimate the expanded uncertainty (k = 2) to be about ±0.3 dB for S_11_ and ±0.5 dB for gain. These values are typical for this type of measurement and are considered acceptable for engineering validation.

**Table 2:** Key dimension of the 2D model for *Allomyrina dichotoma* antennae.

Parameter	Value (mm)	Parameter	Value (mm)	Parameter	Value (°)
W1	10	L1	10	α1	135
W2	0.5	L2	1.7	α2	95
W3	1.3	L3	1.6	α3	90
W4	1.7	L4	1	α4	80
W5	1.3	L5	1.3	α5	25
W6	2	L6	4	α6	30
W7	4	L7	2		

## 3. Results

### 3.1. Performance of the Three-Dimensional Bio-Inspired Dipole Antenna

**Figure 5:**
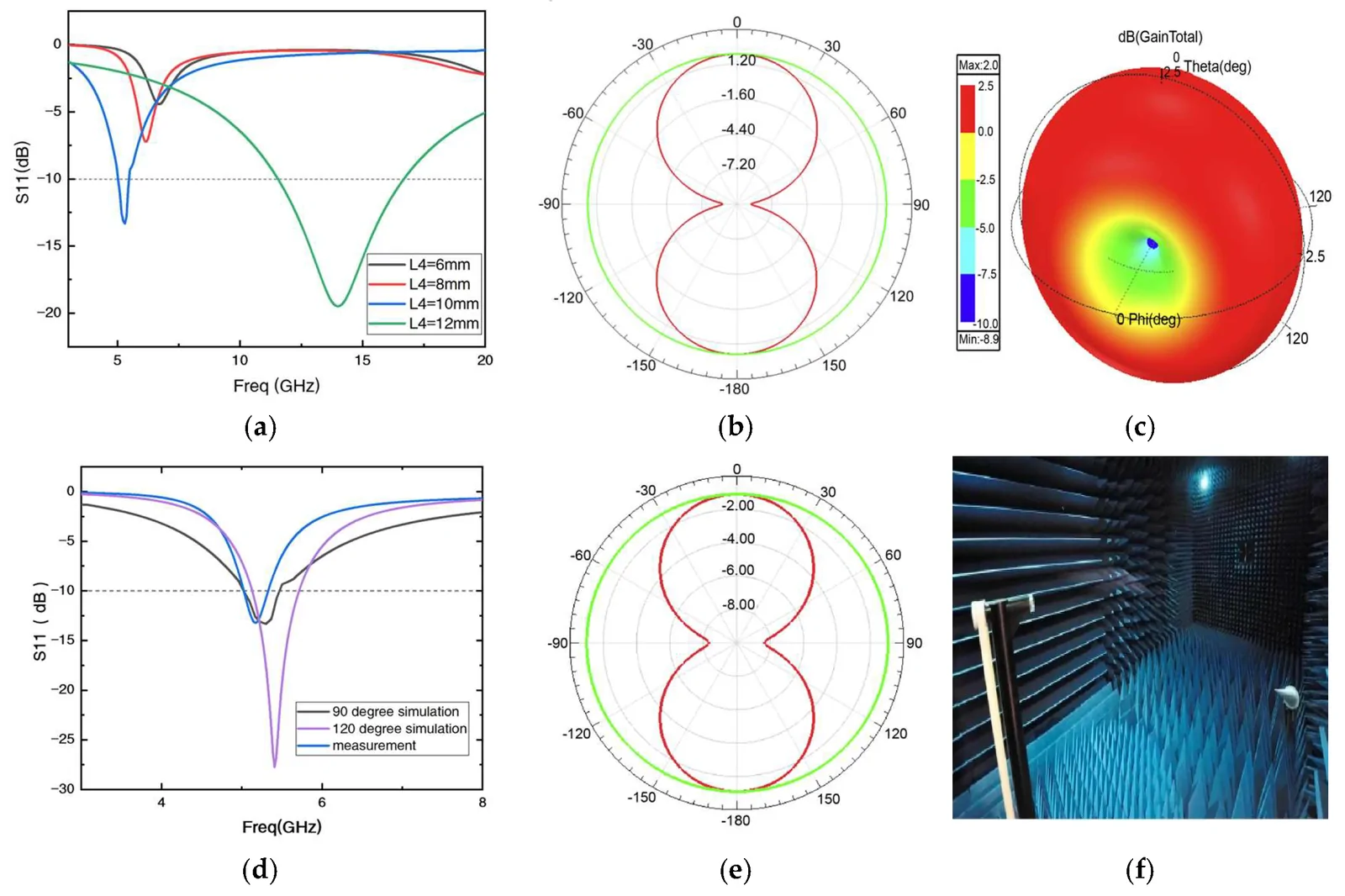
Simulation and measured performance results of the three-dimensional bionic antenna: (**a**) Parametric sweep S11 curves. (**b**) Simulated radiation pattern (*L4* = 10 mm). (**c**) 3D radiation gain plot (*L4* = 10 mm). (**d**) S11 comparison of parametric angle tuning between simulation and measurement. (**e**) Radiation pattern after angle adjustment (120°). (**f**) Photograph of the microwave anechoic chamber test environment.

We first performed full-wave simulations on the 3D bio-inspired dipole antenna based on the established model and setup. Figure 5a shows the simulated S_11_ curves for various feeding-port lengths *L*4. As *L*4 increases from 5 mm to 10 mm, the resonance shifts downward and the impedance matching improves progressively. At 5 mm, a shallow resonance appears around 5.5 GHz (S_11_ ≈ −8 dB); at 7.5 mm, it deepens to about −11 dB at roughly 5.4 GHz; at 10 mm, the antenna achieves S_11_ < −10 dB from 5.03 to 5.47 GHz, with a center frequency of 5.25 GHz and a 440 MHz bandwidth. Further increasing *L*4 beyond 10 mm does not significantly enhance bandwidth but enlarges the antenna beyond the target size. We therefore chose *L*4 = 10 mm as the final feeding dimension.

Under the condition of *L*4 = 10 mm and a lamella-flagellum angle of α = 90°, the simulated gain at the center frequency of 5.25 GHz was 2.0 dBi. Figure 5b shows the three-dimensional radiation pattern at this frequency. The antenna exhibits a typical figure-eight pattern in the azimuth plane, with maximum radiation directed at 0° and 180°, and radiation nulls at ±90°, which is consistent with the radiation characteristics of a dipole antenna. The E-plane pattern exhibits a symmetric dual-lobe structure with a front-to-back ratio better than 15 dB.

To assess the influence of antennal posture on performance, we also simulated the α = 120° configuration. Compared with the 90° case, the center frequency shifts upward by about 0.15 GHz to 5.40 GHz, the bandwidth narrows to approximately 400 MHz, and the directivity decreases slightly. These changes are attributed to the increased tip-to-tip distance between the two arms, which effectively reduces the electrical length. Given its slightly better bandwidth and directivity, along with its closer resemblance to the insect’s resting posture, we adopted the 90° configuration as the baseline for all subsequent three-dimensional designs.

To validate the simulations, we fabricated an antenna sample (*α* = 90°, *L*4 = 10 mm) using pure copper 3D printing and measured its S_11_ through an SMA-connected vector network analyzer. Figure 5c shows the measured S_11_ curve. The measured −10 dB impedance bandwidth extends from 5.02 to 5.36 GHz, with a center frequency of 5.20 GHz, a bandwidth of 340 MHz, and a gain of 1.45 dBi at that frequency. Compared with the simulated values, the center frequency deviates by about 0.05 GHz (1%) and the bandwidth by roughly 100 MHz. The simulated radiation efficiency of the 3D antenna at the center frequency (5.25 GHz) is approximately 70%. We also measured the radiation efficiency at the center frequency, which is 64%. This measured value is lower than the simulation mainly due to the surface roughness and assembly tolerances inherent to metal 3D printing. The result suggests that the 3D antenna maintains a reasonable radiation efficiency despite the fabrication constraints. Overall, the 3D design validates the geometric principle in a shape-preserving form while keeping the efficiency at an acceptable level for proof-of-concept purposes.

Two practical fabrication factors account for the above performance deviations. Primarily, the ±0.1 mm dimensional tolerance of 3D printing creates structural errors, especially on the thin distal branches of the antenna. In addition, soldering processes cannot guarantee strict coplanarity between the two antenna arms. The resulting subtle geometric asymmetry unbalances the differential feed and impairs the antenna’s ideal electromagnetic performance. Overall, the measured data exhibits a consistent variation trend with the simulated counterparts, which validates the feasibility of adopting the proposed multi-segmented antennal model for dipole antenna design.

Notably, the presented antenna achieves an ultra-compact electrical dimension, yielding an electrical length of only 0.17λ at 5.25 GHz. This value is nearly one-third the size of a traditional 0.5λ half-wave dipole. This superior miniaturization performance is enabled by the unique curved multi-segmented structure, which stretches surface current paths substantially longer than the antenna’s actual physical scale. The extended current conduction path effectively increases the equivalent electrical length, demonstrating the potential of multi-segmented bionic structures in electrically small antenna design.

### 3.2. Performance of the Two-Dimensional Bio-Inspired Microstrip Antenna

We first simulated the original non-regularized bionic patch model (Figure 4a,b) to characterize the electromagnetic performance of the original biologically projected profile. The initial structure yielded two valid operating bands of 11.54–12.81 GHz and 24.06–32.77 GHz. However, the Smith chart deviated substantially from the standard 50 Ω impedance point over the full frequency sweep. This indicates that pure segmented biological contours induce non-uniform resonant mode distribution, making continuous low-reflection impedance matching unachievable. Hence, contour regularization and parametric tuning were implemented to homogenize modal distribution and improve broadband matching performance.

**Figure 6:**
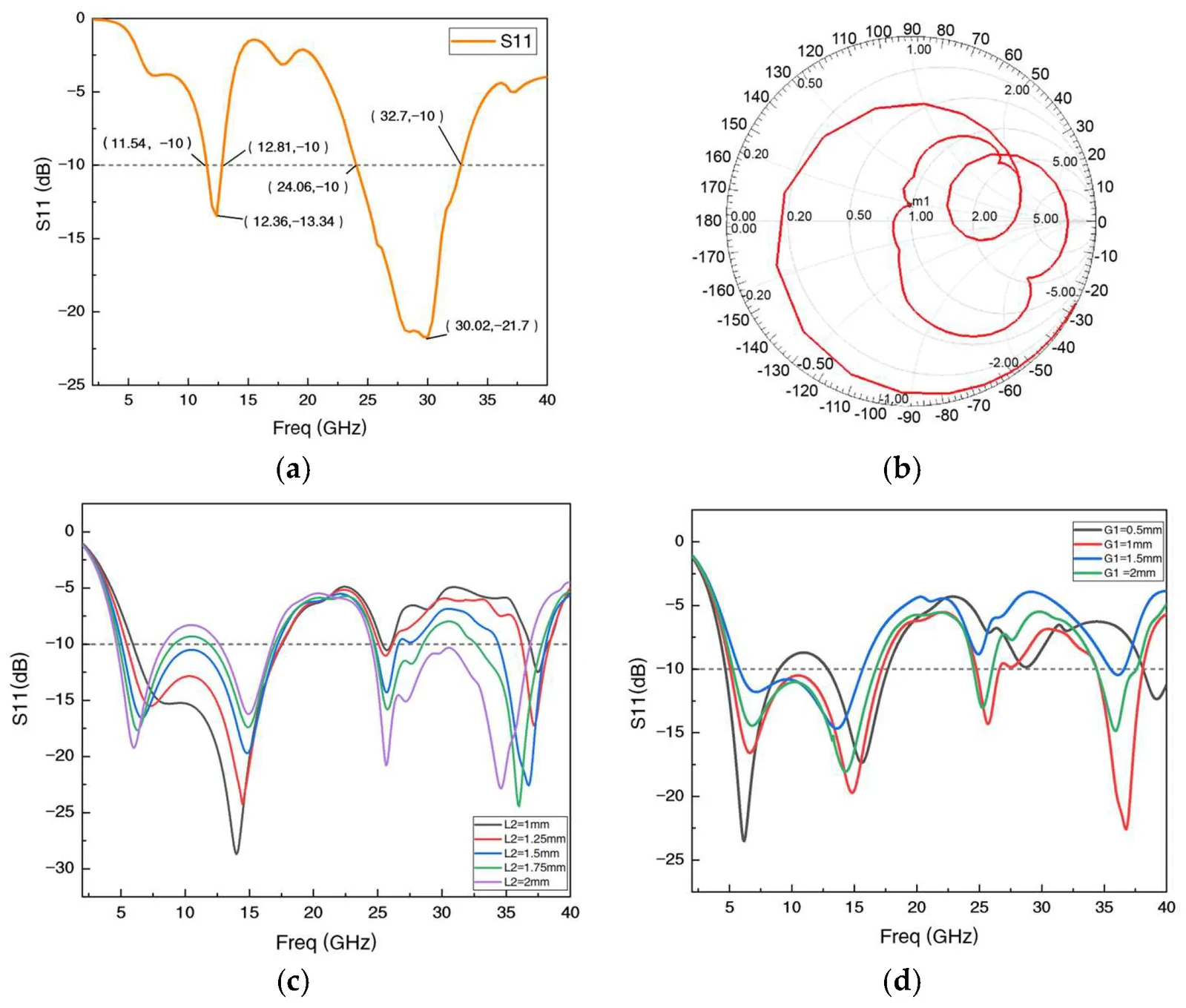
Graphs of the two-dimensional bionic microstrip radiator: (**a**) Simulated S11 curves of the direct projection structure. (**b**) Smith chart of the direct projection structure. (**c**) Parametric tuning results of parameter *L*2 after structural optimization. (**d**) Parametric tuning results of parameter *G*1 after structural optimization.

After structural regularization, key dimensional parameters were swept to optimize antenna performance. Microstrip feed length *L*2 and bottom ground width *G*1 were identified as dominant factors governing impedance matching, with their parametric variations illustrated in Figure 6. Increasing *L2* from 1.0 mm to 1.5 mm significantly optimized high frequency matching above 24 GHz. Further extension to 2.0 mm introduced multiple discontinuous −10 dB attenuation points within the primary 4.86–17.05 GHz working range, destroying bandwidth integrity. Consequently, 1.5 mm was selected as the optimal feed length to maintain stable broadband matching.

For ground width optimization, four candidate values (0.5 mm, 1.0 mm, 1.5 mm, and 2.0 mm) were compared. A width of 1.5 mm provided the optimal solution with minimal S_11_ values and the most continuous frequency coverage. Smaller ground sizes (below 1.0 mm) introduced excessive capacitive effects and deteriorated high-frequency performance, while oversized ground planes (above 2.0 mm) interfered with resonant characteristics and pushed S_11_ beyond the −10 dB threshold in some bands. Therefore, *G1* = 1.5 mm was selected for the prototype design.

**Figure 7:**
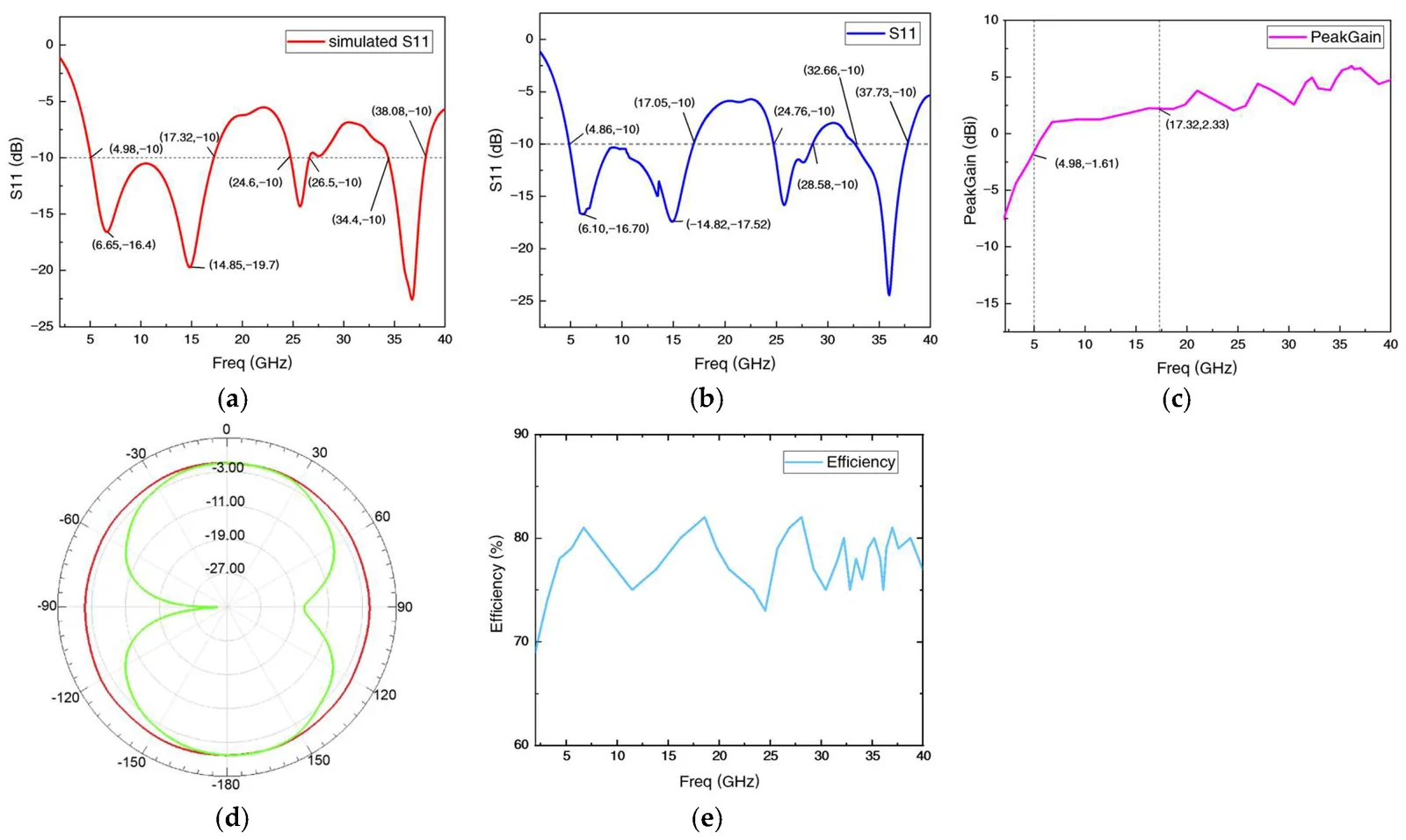
Comparison of simulated and measured performance of the two-dimensional bionic microstrip radiator: (**a**) Simulated S11 curves. (**b**) Measured S11 curves. (**c**) Gain plot. (**d**) Directivity pattern. (**e**) Simulated efficiency curves.

The simulated and measured performance of the optimized planar bionic microstrip antenna is summarized in Figure 7. Simulation results show three effective operating bands where S_11_ remains below −10 dB: 4.98–17.32 GHz, 24.60–26.50 GHz, and 34.40–38.08 GHz. Accordingly, the fabricated prototype achieved measured bandwidths of 4.86–17.05 GHz, 24.76–28.58 GHz, and 32.66–37.73 GHz, showing good consistency with simulation data. Minor frequency deviations are mainly attributed to PCB fabrication tolerances (±0.05 mm) and parasitic effects introduced by SMA soldering, which are within acceptable limits for practical antenna engineering. In the primary band of 4.86–17.05 GHz, the measured fractional bandwidth reaches 111% (center frequency approximately 10.96 GHz). Multiple resonance valleys are observed on the S_11_ curve, located at approximately 6.6 GHz, 10.2 GHz, 14.85 GHz, and 16.5 GHz. These resonance valleys correspond to multiple adjacent resonant modes excited by the individual antennal segments. The mutual coupling and superposition of these modes form a continuous wideband response, consistent with the design expectation described in Section 2.3. In the high-frequency bands of 24.76–28.58 GHz and 32.66–37.73 GHz, S_11_ also remains below −10 dB, indicating that the antenna possesses multi-band capability. Compared with conventional rectangular microstrip patch antennas of the same size (typical bandwidth <10%), the bandwidth of the present design in the primary band is improved by approximately one order of magnitude.

Figure 7c shows the simulated gain across the primary band. From 4.98 to 17.32 GHz, the gain rises steadily from −1.61 dBi to 2.33 dBi and stays around 2.5 dBi in the other two bands, notably higher than conventional patch antennas of similar size. The gain variation with frequency reflects the multi-mode resonance behavior, where changes in current distribution alter the effective aperture. Overall, the gain remains sufficiently high, suggesting that each mode maintains good radiation efficiency. Given the ultra-wideband characteristic and the target 5–17 GHz working range, gain characterization in the anechoic chamber was performed at selected frequency points. Experimental results show that the antenna achieves a peak gain of −2.2 dBi at 4.86 GHz and 2.15 dBi at 17.05 GHz. The great consistency between simulated and measured gain data validates the rationality of the proposed structure and the precision of the numerical antenna model.

Figure 7d shows the E-plane radiation pattern with a red trace, while the green curve corresponds to the H-plane response. Distinct main lobes and side lobes are visible from the plotted radiation patterns. The main lobe, with the highest intensity, is directed along the horizontal axis (0° and 180°). The side lobes appear at approximately −11.00 dB, and the back lobe (at 180° reverse) measures about −27.00 dB. The antenna radiates primarily in the 0° and 180° directions. The cross-polarization level stays below −14 dB, confirming the benefit of the bilaterally symmetric design for polarization purity. The front-to-back ratio is approximately 24 dB, indicating satisfactory suppression of backward interference. Figure 7e presents the simulated radiation efficiency of the 2D antenna across the operating band (4.86–17.05 GHz). The efficiency ranges from approximately 74% to 82%, with an average of about 76% across the entire band. The efficiency remains relatively stable over most of the frequency range, with a slight decrease at lower frequencies near the radiation cutoff. To validate the simulated results, we measured the radiation efficiency at four representative frequencies: 4.86, 6.1, 14.82, and 17.05 GHz. The measured values range from 67% to 72%, slightly lower than the simulated results due to fabrication tolerances and parasitic losses. The comparison confirms that the antenna maintains high radiation efficiency across the operating band. This indicates that the bandwidth enhancement achieved by the multi-segmented irregular structure does not significantly compromise radiation performance.

## 4. Discussion

The engineering results presented in this study can be interpreted from a structural biomimetic perspective and, in turn, inform our understanding of the biological prototype. The lamellate antennal morphology of *Allomyrina dichotoma* has been recognized in biology as the morphological basis for chemoreception. However, far less attention has been paid to the potential versatility of its underlying geometric principle, namely the multi-modal response of a multi-segmented tapered structure under broadband excitation across different physical domains. By mapping this morphology to the electromagnetic domain, we demonstrate that this geometric principle remains effective in a new physical context: the multi-segmented tapered geometry supports broadband multi-mode resonance in the electromagnetic domain, just as it does broadband vibration sensing in the mechanical domain. This finding corroborates, from an engineering standpoint, the structural advantage of this morphology across multiple physical domains. In other words, the evolutionary selection of this morphology as a chemoreceptive apparatus may be partly explained by the inherent versatility of its geometric configuration, independent of the molecular recognition capabilities of the sensilla it carries. Structural biomimetics thus not only contributes to engineering applications but also offers engineering evidence for understanding the evolutionary rationale of biological forms.

### 4.1. Mechanisms of Broadband and Miniaturized Performance in Multi-Segmented Irregular Structures

The two-dimensional bio-inspired microstrip antenna achieves a fractional bandwidth of 111% (4.86–17.05 GHz) within an ultra-compact footprint of 10 × 10 × 1 mm^3^, while the three-dimensional bio-inspired dipole antenna exhibits an electrical length of only 0.17λ. These performance breakthroughs are not incidental but originate from three synergistic mechanisms arising from the multi-segmented irregular structure of the *A. dichotoma* antenna. The three mechanisms are presented in sequence below.

The first mechanism is multi-mode resonant coupling. Given the antenna’s ultra-wideband coverage from 5 to 17 GHz, only selected frequency sampling points were chosen for gain testing inside the anechoic chamber. Measured peak gain reaches −2.2 dBi at 4.86 GHz and 2.15 dBi at 17.05 GHz. The close match between simulated and measured gain data confirms the rationality of the proposed topology and the validity of the simulation model. Distinct dips on the S_11_ curves confirm the excitation of multiple resonant modes. The ten-section bionic antenna works as ten mutually coupled resonators. Sizes taper gradually along the antenna axis, assigning separate resonant frequencies to each unit: broad base segments resonate at low frequencies, while narrow end segments respond to high frequencies. The dips at 6.1 GHz and 14.82 GHz correspond to intrinsic modes formed by electromagnetic coupling between adjacent segments. A broad continuous frequency response can be achieved if adjacent modes are closely spaced, with their frequency gap smaller than half the total bandwidth of the two modes. Each resonant mode here delivers over 1.5 GHz bandwidth, and the interval between modes is roughly 1.8 GHz, satisfying the spectral overlap condition. In short, the wide operating band is not obtained by widening a single resonance, but by stacking numerous coupled resonances generated by this segmented layout.

A second mechanism works alongside multi-mode coupling to realize miniaturization by expanding the antenna’s effective electrical perimeter. Resonant frequency of microstrip patches falls as effective perimeter rises. The winding outlines of the multi-segmented bionic layout lengthen surface current trajectories and enlarge the perimeter. Given identical physical space, such irregular shapes shift resonances downward and cut antenna size at target frequencies. This advantage is more pronounced in 3D curved segmented structures. The antenna only occupies an electrical length of 0.17λ over its linear span, far smaller than the 0.5λ of standard half-wave dipoles.

Balanced radiation aperture serves as the third core working principle, which keeps favorable radiation performance without sacrificing broadband characteristics and compact dimensions. Symmetric bilateral layout restrains the flow of surface currents, as evidenced by measured cross-polarization levels below −14 dB. Such balanced structure aligns the phase of magnetic currents on two radiating edges, enabling constructive superposition of far-field signals and allowing the effective radiation aperture to approach the antenna’s physical aperture. The planar prototype delivers stable gain near 2.15 dBi within its whole working range, while the three-dimensional prototype presents the typical figure-eight radiation pattern of dipoles.

The radiation efficiency results further support the validity of the proposed design. Despite the compact electrical size, the 2D antenna maintains high efficiency across the entire band, indicating that the multi-segmented irregular structure does not introduce significant losses. For the 3D design, the measured efficiency is somewhat lower than the simulated value, mainly due to the surface roughness and assembly tolerances inherent to metal 3D printing. Even so, it remains at an acceptable level for proof-of-concept purposes. These observations confirm that the bandwidth enhancement achieved through the three mechanisms does not come at the cost of radiation performance. They also highlight that fabrication precision plays a critical role in preserving the intrinsic efficiency of the design.

These mechanisms coordinate with each other instead of acting independently. Segmented structures excite multiple resonant modes, tapered curved contours extend the propagation paths of surface currents, and bilateral symmetry stabilizes radiation efficiency. Jointly, they establish a direct correlation between the morphological features of beetle antennae and electromagnetic behaviors. It further explains that the segmented irregular geometry can integrate miniaturization, ultra-wideband coverage, and stable gain into a single antenna design.

### 4.2. Analysis of Bandwidth Discrepancy Between the 2D and 3D Implementations

The two antennas share the same biological prototype, yet their bandwidth performance differs substantially, and this difference can be traced to the distinct implementation pathways chosen.

For the 2D design, we regularized the projected antennal contour. The irregular segments were reorganized into an ordered set of ellipses, semi-ellipses, and rectangles. This sacrifices some morphological accuracy, but it leads to a more even distribution of segment dimensions. As a result, the resonant frequencies become roughly equally spaced in the frequency domain. This kind of spacing helps achieve continuous coverage through multi-mode resonance. For the 3D design, we took a different approach. The original curved morphology was fully preserved, including all local undulations. This makes the model more faithful to the biological template, but it also disturbs the regularity of the resonant mode distribution. The frequency intervals become uneven, which makes it difficult to realize continuous broadband operation.

Fabrication precision is another important factor. The 2D antenna uses standard PCB fabrication with an etching accuracy of ±0.05 mm. This ensures high dimensional fidelity. In contrast, the 3D antenna relies on metal additive manufacturing, which only offers a forming accuracy of ±0.1 mm. For the slender distal segments, the dimensions are already close to the manufacturing limit. Thus, the deviations between the printed geometry and the design become more noticeable. Since multi-mode resonant structures are very sensitive to dimensional errors, any deviation in one segment will shift its resonant frequency. When these errors accumulate, adjacent modes may either separate with gaps or overlap insufficiently, and the bandwidth narrows as a result.

The 3D curvature also affects impedance matching. The 3D design retains the curved lamellate shape, which bends the arms in space. This bending improves compactness, giving an electrical length of only 0.17λ. However, it also introduces extra reactive components at the feed port over a wide frequency range, making matching more difficult. The 2D planar structure avoids this issue and can maintain a stable 50 Ω match across the entire band.

Overall, the 2D design achieves wider bandwidth because it follows a cycle of morphological simplification, orderly mode distribution, and continuous coupling. The 3D design, by trying to preserve the original morphology, ends up sacrificing mode uniformity and impedance smoothness. This trade-off reveals a basic tension between morphological fidelity and electromagnetic performance, which is worth considering in bio-inspired antenna design.

### 4.3. Comparison with State of the Art

The two-dimensional design achieves a fractional bandwidth of 111% across 4.86–17.05 GHz with overall dimensions of only 10 × 10 × 1 mm^3^, which is well suited for highly integrated equipment, including miniature unmanned vehicles and IoT devices. The 3D fabricated version achieves an ultra-short electrical length of 0.17λ, yet suffers degraded bandwidth (66%) and peak gain (1.45 dBi). Such performance differences mainly arise from manufacturing defects: 3D printing has a tolerance of ±0.1 mm, and manual soldering creates angular misalignment between the two radiating arms. By contrast, PCB processing offers tighter precision of ±0.05 mm with negligible geometric distortions. Even with such drawbacks, both prototypes surpass traditional antennas of equivalent volume. This result demonstrates that segmented beetle antenna profiles deliver clear performance advantages whether realized in planar or spatial configurations.

To position our work within the broader literature, Table 3 compares the two- and three-dimensional bio-inspired antennas developed here against representative designs previously reported. Since the cited works differ considerably in their design goals, operating frequencies, and target applications, the comparison focuses on the relative trade-offs between size and bandwidth, rather than a direct ranking of absolute performance metrics.

In the field of two-dimensional bio-inspired antennas, Lozano et al. systematically analyzed the leaf geometries of 100 plant species and applied them as design templates for microstrip patch antennas [12], demonstrating that plant structures can serve as effective bio-inspired tools for antenna design. Building on this foundation, Tan et al. designed a holly-leaf-shaped bio-inspired flexible wideband antenna [13]. By etching vein-like slots into the radiating patch to introduce inter-slot capacitance, they achieved a fractional bandwidth of 100% from 2.4 to 7.1 GHz, with a peak gain of 5.4 dBi. Compared with the work of Tan et al., our two-dimensional design achieves a wider fractional bandwidth (111% vs. 100%) while being substantially more compact [13]. Yu et al., inspired by the spider web structure, designed a flexible multi-band antenna on a 40 × 50 × 0.1 mm^3^ substrate, achieving coverage across three frequency bands [14]. In contrast, our two-dimensional design achieves continuous wideband coverage with an area approximately one-twentieth of that of Yu et al.’s design, rather than discrete multi-narrowband coverage [14]. Omar et al. developed a compact 5G patch antenna based on the serrated structure of beetle antennae, with a footprint of 30 × 30 mm^2^, achieving an absolute bandwidth of 4.69 GHz and a gain of 4.677 dB [25]. Our two-dimensional design achieves approximately eight times the fractional bandwidth (111% vs. about 13.5%) within approximately one-ninth of the area. Nahar et al. designed a miniature bio-inspired antenna based on the sneezewort leaf shape, with dimensions of 18 × 19 × 1.6 mm^3^, a fractional bandwidth of 55.96%, and a peak gain of 2.1 dBi [36]. Our two-dimensional design achieves approximately twice the fractional bandwidth with comparable gain in a smaller footprint.

In the three-dimensional domain, the literature remains sparse. Kyriakou et al. demonstrated the feasibility of directly translating a bat outer-ear morphology into an ultra-wideband antenna, but provided neither physical dimensions nor gain data [19]. In contrast, our three-dimensional design not only validates the same direct transformation from prototype to antenna, but also offers complete dimensional information (~5.6 × 10 × 2.5 mm^3^) and measured performance parameters (0.17λ electrical length, 66% bandwidth, 1.45 dBi gain), making it more quantifiable and reproducible.

We acknowledge that the measured gain at 4.86 GHz is −2.2 dBi, which is lower than the peak gain of 2.15 dBi at 17.05 GHz. This is a direct consequence of the bandwidth–gain trade-off inherent in electrically small antennas: achieving 111% fractional bandwidth within a 10 × 10 × 1 mm^3^ footprint inevitably requires some compromise in gain, particularly at the lower band edge where the electrical size is smallest relative to the wavelength. Nevertheless, this gain level is acceptable for the intended applications. For micro unmanned platforms and IoT terminals, the primary operating bands are in the higher frequency range (X-band and Ku-band), where the gain is consistently above 0 dBi and reaches 2.15 dBi at 17.05 GHz. At the lower band edge, the −2.2 dBi gain still provides sufficient link margin for short-range communication scenarios. More importantly, the compact footprint and 111% bandwidth offer significant advantages in terms of frequency agility and interference resilience. This trade-off is therefore justified by the overall system-level benefits.

The two designs serve different practical purposes. The 2D antenna has a footprint of only 10 × 10 × 1 mm^3^ and offers 111% fractional bandwidth. This makes it a good candidate for micro unmanned platforms and IoT devices. Its operating band covers several commonly used frequency ranges: 5 GHz WIFI and control links, X-band from 8 to 12 GHz for radar sensing, and Ku-band from 12 to 17 GHz for high-speed data downlink. These are the bands needed for small UAVs that combine communication, sensing, and data transmission on a single platform. The ultra-wideband performance also allows frequency hopping, which helps maintain link reliability in congested or contested environments. The antenna uses standard PCB fabrication, which keeps costs low and facilitates mass production for large-scale IoT deployment. The 3D antenna, on the other hand, is more suitable for scenarios where size is the primary constraint. Its electrical length is only 0.17λ, which shows that a biological shape can be directly translated into a working radiating structure without significant modification. The gain and bandwidth of the 3D antenna are lower than those of the 2D version. However, the main contribution here lies in the concept. This design serves as a proof-of-concept that biological forms can be directly translated into radiating structures. It is not intended as a product ready for mass deployment.

In summary, our two-dimensional design is superior or comparable to representative two-dimensional bio-inspired antenna works reported in the literature in both size and fractional bandwidth. The three-dimensional design, given the extremely limited studies on three-dimensional bio-inspired antennas, provides quantifiable design references and experimental data for this direction.

### 4.4. Limitations and Future Work

Although the antennal structure of *Allomyrina dichotoma* has been validated as an effective bionic antenna prototype, this study still contains several limitations worth noting. First, the irregular biological contours of the planar antenna were simplified into standardized ellipses, semi-ellipses, and rectangles during structural regularization. While such processing retains the maximum width and relative positional relationships of each segment, subtle local morphological features are inevitably omitted. Whether these fine geometric details can optimize bandwidth and radiation gain remains to be explored. Second, the fabricated 3D prototype exhibits a discrepancy between simulated and measured performance. The measured gain of 1.45 dBi is lower than the simulated value of 2.0 dBi. This deviation is mainly caused by the ±0.1 mm fabrication tolerance of 3D printing and unavoidable coplanarity errors during soldering, indicating that 3D bionic structures are more sensitive to manufacturing inaccuracies than planar PCB antennas. Moreover, only two discrete angles (90° and 120°) between the lamella and flagellum were investigated in this work, whereas continuous angular variations remain unstudied. Full-range angular parametric analysis is required to clarify angle-dependent electromagnetic characteristics and explore the feasibility of angle-reconfigurable antenna designs.

Correspondingly, future research will focus on three directions to compensate for the above deficiencies. First, quantitative parametric analysis will be carried out to clarify the correlation between contour irregularity and antenna performance, to determine the optimal regularization degree instead of adopting fully regular or completely biomimetic structures. Second, high-precision microscale 3D printing technology will be adopted to reduce fabrication errors and improve radiation gain, while maintaining the ultra-compact electrical size of 0.17λ. Third, exhaustive angular sweeping of the lamella–flagellum structure will be implemented to develop reconfigurable antenna schemes based on mechanical or MEMS angular adjustment.

Furthermore, the research framework established in this work, including biological feature extraction, structural parameterization, electromagnetic analysis, and experimental verification, can be generalized to other insect antenna morphologies, such as long-horned beetles, cicadas, and mosquitoes. Comparative investigations of different bionic structures can construct a complete morphology-performance database, providing abundant innovative design references for future bio-inspired antenna development.

## 5. Conclusions

This study demonstrates that the multi-segmented tapered geometry of the *Allomyrina dichotoma* antenna can be extracted as a structural biomimetic principle and mapped to microwave antenna design. The 2D design achieves 111% fractional bandwidth from 4.86 to 17.05 GHz with a peak gain of 2.15 dBi, while maintaining a radiation efficiency of 67–72% across the operating band. The 3D design validates the same geometric principle in a shape-preserving configuration, achieving an electrical length of only 0.17λ. These results suggest that the 2D design is well suited for practical applications such as micro unmanned platforms and IoT terminals, where compact size and broadband performance are both important. The 3D design, although less practical for direct deployment, offers value for proof-of-concept studies and applications that require extreme miniaturization. Several limitations should be noted. The 2D design involves simplifying the irregular contours, which may sacrifice some subtle electromagnetic effects, and the 3D design is limited by the fabrication accuracy of metal 3D printing. Future work could focus on finding the optimal level of contour irregularity, improving fabrication precision, and extending this approach to other insect species to build a more comprehensive morphology-performance database.

## Figures and Tables

**Table 1:** Key dimension of the 3D model for *Allomyrina dichotoma* antennae.

Parameter	Value (mm)	Parameter	Value (mm)
L1	2.9	H4	0.4
L2	0.6	H5	0.3
L3	0.7	H6	0.4
H1	1.4	H7	0.4
H2	0.2	H8	0.9
H3	0.3	H9	1.8

**Table 3:** Performance comparison between the proposed bionic antenna and existing bionic antenna designs.

Reference	Bionic Prototype	Fabrication Complexity	Size (mm^3^)	Operating Band (GHz)	Impedance Bandwidth	Peak Gain (dBi)	Application Scenarios
[12]	Plant leaves (100 species, 2D)	-	-	2.4/5 (Dual-band)	-	-	-
[13]	Holly leaf (2D)	FPC	-	2.4–7.1	100% (Fractional Bandwidth)	5.4	low detectability application
[14]	Spider web (2D)	FPC	40 × 50 × 0.1	1.31–2.0/3.4–4.0/5.1–5.78	42.4%/16.2%/12.5%	-	smart wireless devices
[25]	Sawtooth-shaped beetle antennae (2D)	PCB	30 × 30 (Area)	Single resonance at 30.5 GHz	4.69 GHz (Absolute Bandwidth)	4.677	5G mobile communications
[36]	*Oxalis corniculata* leaf (2D)	PCB	18 × 19 × 1.6	3.16–5.42	55.96% (Fractional Bandwidth)	2.1	Sub-6 GHz Consumer Wireless and Biomedical Diagnostic Applications
[19]	Bat pinna (3D)	-	-	UWB	-	-	-
**This work (2D)**	**Two-horned rhinoceros beetle antennae**	**PCB**	**10 × 10 × 1**	**4.86–17.05**	**111% (Fractional Bandwidth)**	**2.15**	**micro unmanned platforms and IoT terminals**
**This work (3D)**	**Two-horned rhinoceros beetle antennae**	**Metal 3D** **printing**	**5.6 × 10 × 2.5**	**5.02–5.36**	**6.8% (Fractional Bandwidth)**	**1.45**	**proof of concept**

## Data Availability

The data presented in this study are available upon request from the corresponding author.
